# Mensenchymal stem cells can delay radiation-induced crypt death: impact on intestinal CD44^+^ fragments

**DOI:** 10.1007/s00441-015-2313-6

**Published:** 2015-11-27

**Authors:** Peng-Yu Chang, Xing Jin, Yi-Yao Jiang, Li-Xian Wang, Yong-Jun Liu, Jin Wang

**Affiliations:** Department of Radiation Oncology, The First Bethune Hospital of Jilin University, Changchun, 130021 People’s Republic of China; Changchun Institute of Applied Chemistry, Chinese Academy of Sciences, Changchun, 130000 People’s Republic of China; Ever Union Biotechology, Tianjin, 300162 People’s Republic of China; Department of Cardiac Surgery, TEDA International Cardiovascular Hospital, Tianjin, 300000 People’s Republic of China; Tianjin Institute of Industrial Biotechnology, Chinese Academy of Sciences, Tianjin, 300300 People’s Republic of China; Alliancells Bioscience, Tianjin, 300300 People’s Republic of China

**Keywords:** Mesenchymal stem cell, CD44, Radiation-induced death, Organoids, Crypt domains

## Abstract

Intestinal stem cells are primitive cells found within the intestinal epithelium that play a central role in maintaining epithelial homeostasis through self-renewal and commitment into functional epithelial cells. Several markers are available to identify intestinal stem cells, such as Lgr5, CD24 and EphB2, which can be used to sort intestinal stem cells from mammalian gut. Here, we identify and isolate intestinal stem cells from C57BL/6 mice by using a cell surface antigen, CD44. In vitro, some CD44^+^ crypt cells are capable of forming “villus-crypt”–like structures (organoids). A subset strongly positive for CD44 expresses high levels of intestinal stem-cell-related genes, including *Lgr5*, *Bmi1*, *Hopx*, *Lrig1*, *Ascl2*, *Smoc2* and *Rnf43*. Cells from this subset are more capable of developing into organoids in vitro, compared with the subset weakly positive for CD44. However, the organoids are sensitive to ionizing irradiation. We investigate the specific roles of mesenchymal stem cells in protecting organoids against radiation-induced crypt death. When co-cultured with mesenchymal stem cells, the crypt domains of irradiated organoids possess more proliferative cells and fewer apoptotic cells than those not co-cultured with mesenchymal stem cells. *Cd44v6* continues to be expressed in the crypt domains of irradiated organoids co-cultured with mesenchymal stem cells. Our results indicate specific roles of mesenchymal stem cells in delaying radiation-induced crypt death in vitro.

**Electronic supplementary material:**

The online version of this article (doi:10.1007/s00441-015-2313-6) contains supplementary material, which is available to authorized users.

## Introduction

In mammals, the intestinal epithelium represents a tissue that self-renews for an entire lifetime. Typically, the intestinal epithelium requires a period of 4–5 days for one complete turnover (van der Flier and Clevers 2009). Homeostasis in the intestinal epithelium is accurately controlled by the proliferation of intestinal stem cells (ISCs) and their commitment into functional epithelial cells.

Currently, two pools of ISCs are acknowledged to occur within the crypts (Li and Clevers 2010). Herein, the Lgr5^+^ ISCs located at the basement of crypts (termed “crypt base columnar [CBC] stem cells”) represent a population of cells that maintain epithelial homeostasis under intact conditions (Barker et al. 2007). The exact duration of cell-cycling in CBC stem cells is 21.5 h (Schepers et al. 2011). When dividing, the parental DNA strands in the CBC stem cells are randomly segregated into their progeny (Schepers et al. 2011). Some cells will competitively bind to the surfaces of Paneth cells to keep their “stemness” (Snippert et al. 2010). The rest, without provisions from Paneth cells, will develop into transit-amplifying (TA) progenitors that will replenish the apoptotic cells by differentiating into functional cells along the villus-crypt axis (Snippert et al. 2010). In contrast to CBC stem cells, slow-cycling ISCs (termed “reserve ISCs”), which are positive for Bmi1, Lrig1, Hopx, or mTERT at the 4+ position of the crypts, represent a population of cells that undertake epithelial reversion upon intestinal injuries through their direct commitment to functional epithelial cells or through their conversion into CBC stem cells (Sato and Clevers 2013; Tian et al. 2011). Similarly, Dll1^+^ TA progenitors are capable of reprogramming themselves into CBC stem cells in order to promote epithelial regeneration (van Es et al. 2012).

However, any stimuli altering the genomic stability of ISCs will interrupt the epithelial homeostasis. For example, mutation in the *Apc* gene will lead to persistent activation of β-catenin/Tcf signaling pathway, resulting in a wild proliferation of CBC stem cells and subsequent neoplastic formation in the gut (Morin et al. 1997). Moreover, the deletion of thymine-guanine in the 3′ untranslated region of *Cd24* gene in ISCs contributes to increased susceptibility to Crohn’s disease (Van Limbergen et al. 2015). Thus, an investigation of ISC characteristics should improve public awareness of the pathogenesis of such diseases. In this context, Sato et al. (2009) first established a three-dimensional (3D) culture system that mimicked the development of CBC stem cells in vivo; one single CBC stem cell was capable of forming into a “villus-crypt”-like structure (termed “organoids” below). Moreover, these organoids can be repeatedly expanded for up to 1 year (Sato et al. 2009). Based on these encouraging data, two studies were separately carried out to evaluate the therapeutic potentials of organoids on epithelial injuries in colon (Jung et al. 2011; Yui et al. 2012). The results showed that these organoids contributed significantly to epithelial regeneration, which depended on their long-lived potential to repair injured epithelium (Jung et al. 2011; Yui et al. 2012). Hence, regenerative therapy involving the use of ISCs will be an alternative option for managing intestinal injuries (Sato and Clevers 2013).

Nowadays, C57BL/6^lgr5-eGFP-IRES-CreERT2^ reporter mice are the most popular sources for isolating CBC stem cells. Furthermore, some wild-type hosts are still an option for the isolation of ISCs. For example, the surface antigens CD24 or EphB2 have been reported to be candidates for the isolation of ISCs from murine or human gut (von Furstenberg et al. 2011; Sato et al. 2011a). Additionally, ISCs are reported to exist in the side-population (SP) of epithelial cells, as indicated by scatter diagrams obtained by using the fluorescence-activated cell sorting (FACS) technique (von Furstenberg et al. 2014). In addition to these encouraging results, some evidence suggests that the *Cd44* gene is a target of the Wnt/β-catenin signaling pathway responsible for proliferation in CBC stem cells and the maturation of Paneth cells (van der Flier and Clevers 2009; Zeilstra et al. 2008, 2014; Wielenga et al. 1999). On this basis, we speculated that CBC stem cell proliferation will be accompanied by high levels of *Cd44* gene expression. To test this hypothesis, we attempted to isolate ISCs from wild-type mice (strain: C57BL/6) by using CD44 antibody. Our results primarily showed that ISCs existed with crypt cells which had a high expression of *Cd44*, indicating that CD44 is a candidate for marking ISCs in murine gut. Moreover, the CD44^+^ ISCs resembled CBC stem cells. In addition, our previous findings suggested that the infusion of human adipose-derived mesenchymal stem cells (hAd-MSCs) could accelerate epithelial regeneration in irradiated intestine (Chang et al. 2013). In the present study, hAd-MSCs were used to investigate their specific roles in minimizing epithelial injuries through the co-culture of these cells with organoids. Relevant results showed that MSCs were capable of delaying radiation-induced crypt death.

## Materials and methods

### Mice

Male juvenile C57BJ/6 mice (6 weeks old) of an inbred strain and specific pathogen-free (SPF) level were provided by the Laboratory Animal Institute of the Chinese Academy of Military Medical Science (Beijing, China). The mice were housed in an authorized SPF-level animal room of Alliancells Bioscience (Tianjin, China). The study protocols were consistent with good animal practices under approval from our local animal care and use committee.

### Crypt fragment isolation and culture

As described by Sato et al. (2009), the whole small intestine was isolated from C57BJ/6 mice and was split in a longitudinal direction. The intestine was washed repeatedly in pre-chilled D-Hanks buffer (pH 7.4) to remove feces. The villi were then slightly scraped by using a sterilized coverslip and the intestinal tissue was cut into small pieces. The fragments were placed into a 50-ml tube (Corning, USA) and washed with pre-chilled D-Hanks buffer (pH 7.4). Once the fragments had settled down to the bottom of the tube, 30 ml isolation-buffer (2 mM EDTA in D-Hanks buffer) was added to the tube to suspend the fragments once again. Thereafter, the fragments were incubated on ice for 30 min with rotation. After incubation, the supernatant was removed and 30 ml pre-chilled D-Hanks buffer (pH 7.4) was added to the tube with agitation in order to release the crypt fragments. The suspension was then passed through a 70-μm strainer (BD Bioscience, Franklin Lakes, N.J., USA) and the filtrate was collected in a 1 % bovine serum albumin (BSA)-coated 50-ml tube (Corning). Subsequently, the suspension was centrifuged at 1000 rpm for 5 min and the supernatant was removed carefully. Unavoidably, blood cells or adipocytes were contained within the collected crypt fragments. To exclude them by their weights, the crypt fragments had to be washed with 10 ml basal culture medium (BCM) and then centrifuged at 500 rpm for 2 min in a 15-ml tube (Corning, USA) two or three times. After centrifugation, the crypt fragments at the bottom of the tubes were of high purity. These fragments were carefully suspended by using growth-factor-reduced phenol-red-free Matrigel (BD Bioscience) at a ratio of 500 crypts per 50 μl Matrigel. All procedures were processed on ice to prevent the Matrigel from heat-induced solidification. Thereafter, 50 μl Matrigel containing crypt fragments were added into each well of a 24-well plate in order to create circles of Matrigel at 37 °C. Then, 500 μl crypt culture medium (CCM) was supplied to each well for culture at 37 °C under a humid atmosphere with 5 % CO_2_. The medium was changed every 4–6 days. Of note, CCM should be made 30 min before crypt plating. The CCM contained 1× Glutamax (Invitrogen, USA), 1× HEPES (Invitrogen), 100 U/ml penicillin (Invitrogen), 100 μg/ml streptomycin (Invitrogen), 1 × N2 (Invitrogen), 1× B27 (Invitrogen), 1 mM N-acetylcysteine (Sigma-Aldrich, USA), 100 ng/ml epithelial growth factor (EGF; Peprotech, USA), 100 ng/ml Noggin (Peprotech, USA) and 1000 ng/ml R-spondin (Peprotech, USA) in Advanced-DMEM/F12 medium (Invitrogen). The BCM contained 1× Glutamax (Invitrogen), 1× HEPES (Invitrogen), 100 U/ml penicillin (Invitrogen) and 100 μg/ml streptomycin (Invitrogen) in Advanced-DMEM/F12 medium (Invitrogen). The reagents were stored according to the manufacturers’ instructions.

### Isolation and culture of CD44^+^ ISCs

As mentioned above, the isolated crypts were incubated in 1 ml single-cell released medium (SRM; BCM plus 1× N2, 1× B27, 10 μM Y-27632 dihydrochloride, 0.1 % BSA) for 30 min at room temperature with frequent rotation to release the cells. Importantly, repeated pipetting every 10 min was essential to avoid the formation of cell-clusters during incubation. Subsequently, the cell suspension was passed through a 40-μm strainer (BD Bioscience) and then though a 20-μm strainer (Millipore). The filtrate was collected for further washes in SRM (3 times).

During incubation for single cell releasing, rabbit anti-mouse CD44 primary antibody (Santa Cruz Biotechnology, Santa Cruz, Calif., USA) was added to SRM at the ratio of 1:50 (w/v). After a filtering step, the cells were washed in SRM (3 times) to remove unconjugated primary antibody. Thereafter, the number of viable single-cells was determined and microbead-conjugated goat anti-rabbit IgG secondary antibody (Milteny) was diluted in SRM at a ratio of 1:4 (v/v). The cells were incubated in SRM containing 20 % secondary antibody on ice for 15 min and then passed through the separator column. The filtrate was discarded and the cells in the separator column were collected (Positive selection). Batches each with 100 single-cells were suspended in 10 μl Matrigel containing 1 μM Jagged-1 (Ana Spec) and were plated on 96-well plates supplemented with 100 μl intestinal stem cell culture medium (ICM) containing 10 μM Y-27632 dihydrochloride (Sigma-Aldrich, USA) and 100 ng/ml Wnt-3a (Peprotech, USA) diluted in CCM. Cytokines were supplied every 2 days after the cells had been plated and the medium was completely changed every 4–6 days. The Y-27632 dihydrochloride and Wnt-3a peptide were present only during the first 6 days of cell culture. When Paneth cells were observed under an inverted microscope, the ICM could be seen to be completely replaced by CCM.

### Fluorescence-activated cell sorting

To analyze the phenotype of the CD44^+^ crypt cells, rat anti-mouse CD31-phycoerythrin (PE), CD34-PE, CD44-allophycocyanin (APC) and CD45-APC antibodies were used. IgG2b-APC and IgG2a-PE were employed as isotypes. All antibodies were purchased from eBioscience (San Diego, Calif., USA). All experimental procedures were performed in accordance with the manufacturer’s instructions. In addition, the CD44^-^, CD44^hi+^ and CD44^low+^ cells were separately sorted by using the FACS method on a MoFlo XDP (Beckman Coulter, Brea, Calif., USA).

### Histological analysis

Immunohistochemical (IHC) staining was used to determine the distribution of Lgr5^+^ ISCs and CD44^+^ cells and of the Ki67^+^ proliferative cells in the intestinal epithelium. In detail, paraffin-embeded sections were examined by IHC staining. First, sections (4 μm) were dewaxed and rehydrated in dimethylbenzene followed by a gradient of alcohol. Then, the sections were immersed in 0.3 % hydrogen peroxide solution to block endogenous peroxides. Next, antigen retrieval was carried out in 1× sodium citrate solution. Thereafter, sections were incubated with goat serum for 30 min at room temperature to block non-specific antigen-binding sites. Before this step, if the antigen was expressed in the nucleus, the cell membranes in sections had to be ruptured by incubation with 0.25 % Triton X-100 (Roche, Basel, Switzerland) solution for 10 min at room temperature. Primary antibodies, including rabbit anti-mouse Lgr5 (Santa Cruz Biotechnology, Santa Cruz, Calif., USA), rabbit anti-mouse CD44 (Santa Cruz) and rabbit anti-mouse Ki67 (Abcam, Cambridge, Mass., USA), were diluted according to the manufacturers’ instructions. The diluted primary antibodies were incubated with sections overnight at 4 °C. Thereafter, goat anti-rabbit IgG secondary antibody (Abcam) was used for detecting positive cells in the section after an incubation time of 2 h at 37 °C. The cell nucleus was stained by using hematoxylin (Sigma-Aldrich). Imaging was carried out by means of a BX51WI microscope (Olympus, Japan).

Immunocytochemical (ICC) staining was used to evaluate the distribution of CD44^+^ cells and Ki67^+^ proliferative cells in organoids and the differentiation of CD44^+^ ISCs into epithelial cells. In detail, the organoids were incubated with 4 % paraformaldehyde solution for 30 min at 37 °C, mechanically removed from Matrigel into an EP tube (Corning) and incubated with blocking solution for 30 min at room temperature. Before this step, if the antigens were expressed in the nucleus, 0.25 % Triton X-100 (Roche) solution was used for permeabilization for 5 min at room temperature. Diluted primary antibodies were added to the EP tubes at the concentrations recommended by manufacturers. Incubation was carried out overnight at 4 °C. After the primary antibodies had been discarded and following two to three washes in phosphate-buffered saline (pH 7.4), diluted secondary antibodies were added to the EP tubes. The incubation duration was 2 h at 37 °C. The cell nuclei were stained with 4,6-diamidino-2-phenylindole (DAPI; Invitrogen). Imaging was carried out by means of an SP5-II confocal microscope (Leica, Germany).

Transferase-mediated deoxyuridine triphosphate-biotin nick end labeling (TUNEL)-staining was used to detect apoptotic cells, both in vitro and in vivo. The in situ cell death detection kit, POD (Roche), was employed in the present study. All experimental processes were performed according to the manufacturer’s instructions. Imaging was carried out separately by means of a BX51WI microscope (Olympus, Japan) and an SP5-II confocal microscope (Leica, Germany).

Transmission electron microscopy (HITACHI, Japan) was used to analyze the subcellular structures of CD44^+^ISC-derived organoids. The organoids were fixed with 2.5 % glutaraldehyde for 2 h. Thereafter, dehydration of the samples was carried out via a gradient of acetone solutions. The dehydrated organoids were embedded in Epon 812 and sectioned at a thickness of 50 nm. The sections were stained with 5 % uranyl acetate, lead citrate and 3 % potassium phosphotungstate.

### Chemotaxis of MSCs

MSCs from human adipose tissue were purchased from SciencCell Research Laboratories (Carlsbad, Calif., USA). To investigate the specific roles of MSCs on irradiated organoids, a co-culture system was established. The crypt fragments were cultured for 7 days in a 12-well transwell plate before receiving irradiation. When the crypt fragments developed into organoids, on the 7th day the organoids were irradiated by using an RS-2000 Pro Biological Irradiator (Rad-Source, Suwanee, Ga., USA). Immediately, MSCs of passage 6 were plated onto the upper mesh of 12-μm bore diameter. Before this step, the MSCs had been labeled by using CM-Dil cell tracker (Invitrogen), a lipophilic marker, according to the manufacturer’s instructions.

### Real-time polymerase chain reaction

Semi-quantitative reverse transcription (RT) followed by the polymerase chain reaction (PCR) was used to evaluate ISC-related gene expression by the CD44^-^, CD44^low+^ and CD44^hi+^ subpopulations. The total RNA in 5 × 10^5^ of sorted cells was extracted by using an RNA Extraction Kit (Takara-bio, Shiga, Japan). Thereafter, 1 μg total RNA from each sample was used for the synthesis of first-strand cDNA by using an RT-PCR Kit (Takara). Total cDNA was then added to primers for mouse *Lgr5*, *Bmi1*, *Lrig1*, *mTert*, *Hopx*, *Ascl2*, *Rnf43*, *Smoc2* and *Prominin-1*. Additionally, mouse actin primers were used as controls. The sequences of these primers are listed in Supplemental Table S1. Semi-quantitative PCRs were performed by using SYBR Green I Taqman probes (Roche) in 40 amplifying cycles in ABI 7500 Fast equipment (Beckman Coulter).

RT-PCR was used for comparing the *Cd44v6* expression levels of irradiated organoids with or without MSC intervention. All experimental procedures were in accordance with the above information. The sequences of primers for *Cd44v6* are listed in Supplemental Table S1.

### Statistical analysis

Data were analyzed by using SPSS 17.0 software (SPSS, Chicago, Ill., USA) and are shown as means ± standard deviation (SD). The paired *t*-test was performed to compare data between two groups. Statistical significance was defined as *P* ≤ 0.05.

## Results

### CD44^+^ cells are mainly located at putative positions of ISCs within crypts

ISCs are known to be uniformly located within the crypt domain in mammals. The determination of ISCs in vivo relies on two rules: the expression of the ISC-related marker and location at putative positions of ISCs in crypts (Clevers 2013). For example, the CBC stem cells highly express the Wnt-targeted gene, *Lgr5* and are located between two Paneth cells (Barker et al. 2007). Meanwhile, some Lgr5^+^ ISCs are also located at the 4+ position of the crypt (Barker et al. 2007).

**Fig. 1:**
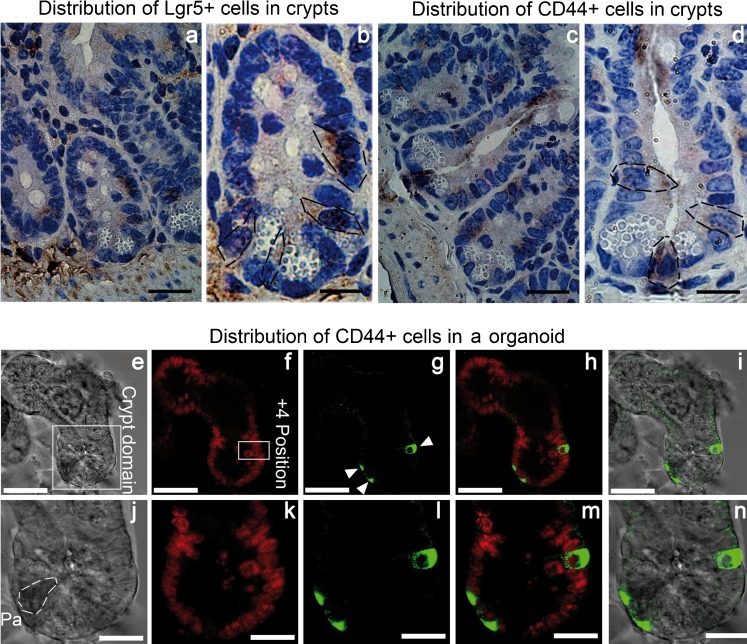
Distribution of CD44^+^ cells within intestinal epithelium. **a**, **b** Immunohistochemical (IHC) staining for Lgr5^+^ ISCs (*black dotted lines*) in vivo. **c**, **d** IHC staining for CD44^+^ cells (*black dotted lines*) in vivo. **a**, **c** Magnification ×400. *Bars* 50 μm. **b**, **d** Magnification ×1000. *Bars* 20 μm. **e–n** Immunocytochemical (ICC) staining for CD44^+^ cells in vitro. **e**, **j** Differential interference contrast (DIC) imaging. **f**, **k** Propidium iodide (PI) staining for nuclei. **g**, **l** Fluorescein isothiocyanate (FITC) for CD44^+^ cells (*white arrowheads* crypt cells strongly positive for CD44). **h**, **m** Overlay of PI image and FITC image. **i**, **n** Overlay of FITC image and DIC image. **e–i** Magnification ×200. *Bars* 200 μm. **j–n** Magnification ×630. *Bars* 100 μm

To determine the specific distribution of CD44^+^ putative ISCs in the crypts, the Lgr5^+^ ISCs were set as positive controls (Fig. 1a, b). As shown in Fig. 1c, d, some cells that were located at the crypt basement and intermingled with Paneth cells (containing granules in plasma) were strongly positive for CD44. The in vitro study also indicated that the cells positive for CD44 were mainly located at the crypt basement, in addition to the 4+ position (Fig. 1e–n). Since the CD44^+^ cells were mainly located at the putative positions of ISCs within the crypt, we speculated that the ISCs existed in the population of CD44^+^ crypt cells.

### One population of ISCs exists in CD44^+^ crypt cells

**Fig. 2:**
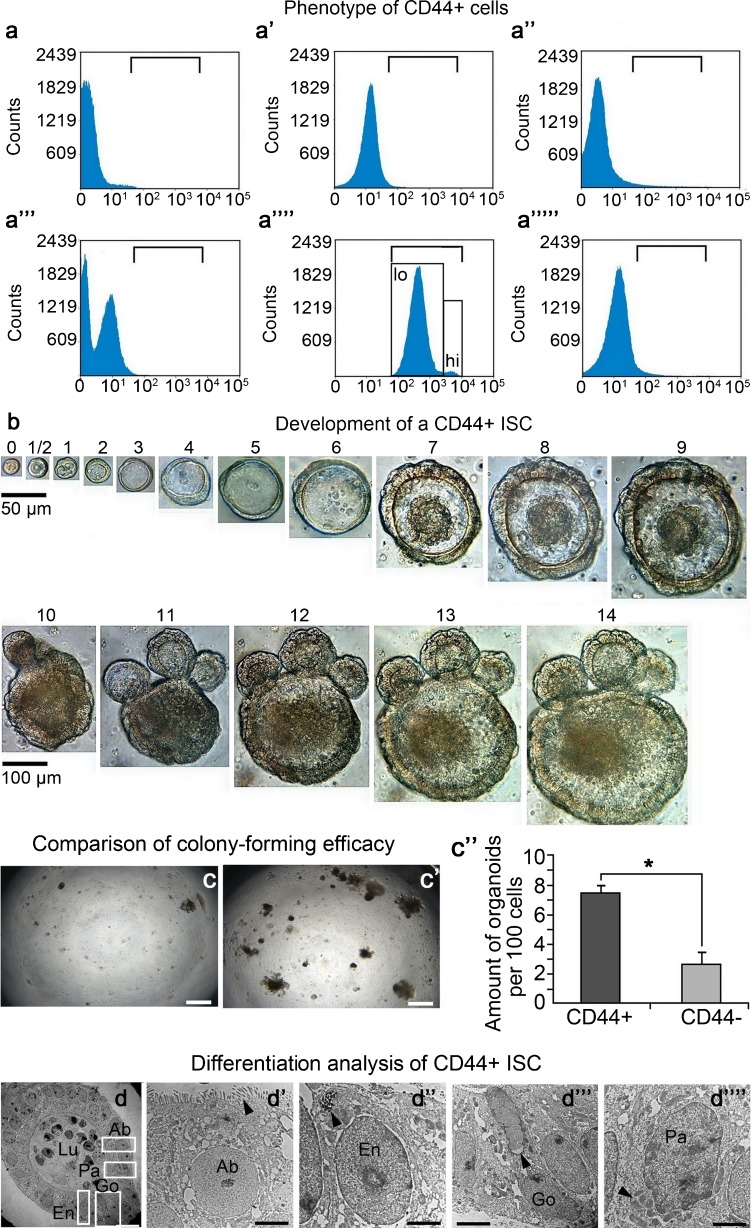
Identification of CD44^+^ cells. **a–a’’’’’** Fluorescence-activated cell sorting (FACS) analysis for cellular phenotype. **a** Isotype control, IgG2a-phycoerythrin (PE). **a’** CD31-PE. **a’’** CD34-PE. **a’’’** Isotype control, IgG2b-allophycocyanin (APC). **a’’’’** CD44-APC (*lo* low-positive for CD44, *hi* high-positive for CD44). **a’’’’’** CD45-APC. **b** Development of CD44^+^ ISC in 3D-culture system. *Numbers* represent days. *Top* Magnification ×400. *Bar* 50 μm. *Bottom* Magnification ×200. *Bars* 100 μm. **c–c’’** Colony-forming efficacies of CD44^-^ cells and CD44^+^ cells. **c** CD44^-^ cells in 3D-culture system for 14 days. **c’** CD44^+^ cells in 3D-culture system for 14 days. **c**, **c’** Magnification ×40. *Bars* 500 μm. **c’’** Comparision of colony-forming efficacy per 100 sorted cells seeded in one well of a 96-well plate. CD44^-^ group in 48 wells; CD44^+^ group in 48 wells. Data represent means ± SD of 48 independent measurements (*n* = 48). *Bars* SD values. Paired *t*-test was used for data analysis. *P ≤ 0.05 represents high significance (CD44^+^ group versus CD44^-^ group). All experimental procedures were repeated twice. **d–d’’’’** Transmission electron microscope imaging of CD44^+^ ISC differentiation. CD44^+^ ISC were cultured in the 3D-system for 6 days and formed a cystic structure. **d** Cystic structure of a single CD44^+^ ISC-derived organoid at 6 days. *Boxed areas* are shown at higher magnification in **d’–d’’’’** (*Lu* lumen). Magnification ×400. *Bar* 50 μm. **d’** Absorptive cell (*Ab*). **d’’** Endocrine cell (*En*). **d’’’** Goblet cell (*Go*). **d’’’’** Paneth cell (*Pa*). *Black arrowhead* in **d’** indicates brush border. *Black arrowheads* in **d’’–d’’’’** indicates granules. **d’–d’’’’** Magnification ×1500. *Bars* 5 μm

To test the above hypothesis, we first isolated the CD44^+^ crypt cells from the small intestine by using a microbead-based sorting technique. FACS analysis showed that the purity of these sorted cells was extremely high (∼99 %; Fig. 2a’’’’). The results of the phenotypic analysis showed that these CD44^+^ crypt cells were negative for CD31, CD34 and CD45, indicating that these cells did not originate from the endothelial and hemapoietic lineages (Fig. 2a–a’’’’’). Next, we cultured these cells in our 3D-system. The CD44^-^ crypt cells were set as controls. Compared with the CD44^-^ controls, some CD44^+^ cells were seen to be able to form organoids after being cultured for 2 weeks (Fig. 2b). The colony-forming efficacy of CD44^+^ cells was significantly higher than that of the CD44^-^ controls with an increase of approximately   three-fold (Fig. 2c–c’’), indicating that the ISCs mainly consisted of a CD44^+^ population.

Next, we wished to determine whether the CD44^+^ crypt cells, which can develop into organoids, were ISCs. Of note, the “stemness” of the tissue-specific stem cells is defined as their rapid self-renewal and multi-lineage differentiation (Clevers 2013). By tracing the development of some CD44^+^ crypt cells, we found that these cells constantly expanded their numbers during a period of 2 weeks (Fig. 2b), indicating that these cells were capable of dividing. Moreover, the CD44^+^ crypt-cell-derived organoids contained all the functional cells of the epithelium of the small intestine, including absorptive cells, endocrine cells, goblet cells and Paneth cells, indicating that these CD44^+^ crypt cells were ISCs (Fig. 2d–d’’’’, Supplemental Fig. S1).

### CD44^+^ ISCs resemble CBC stem cells

**Fig. 3:**
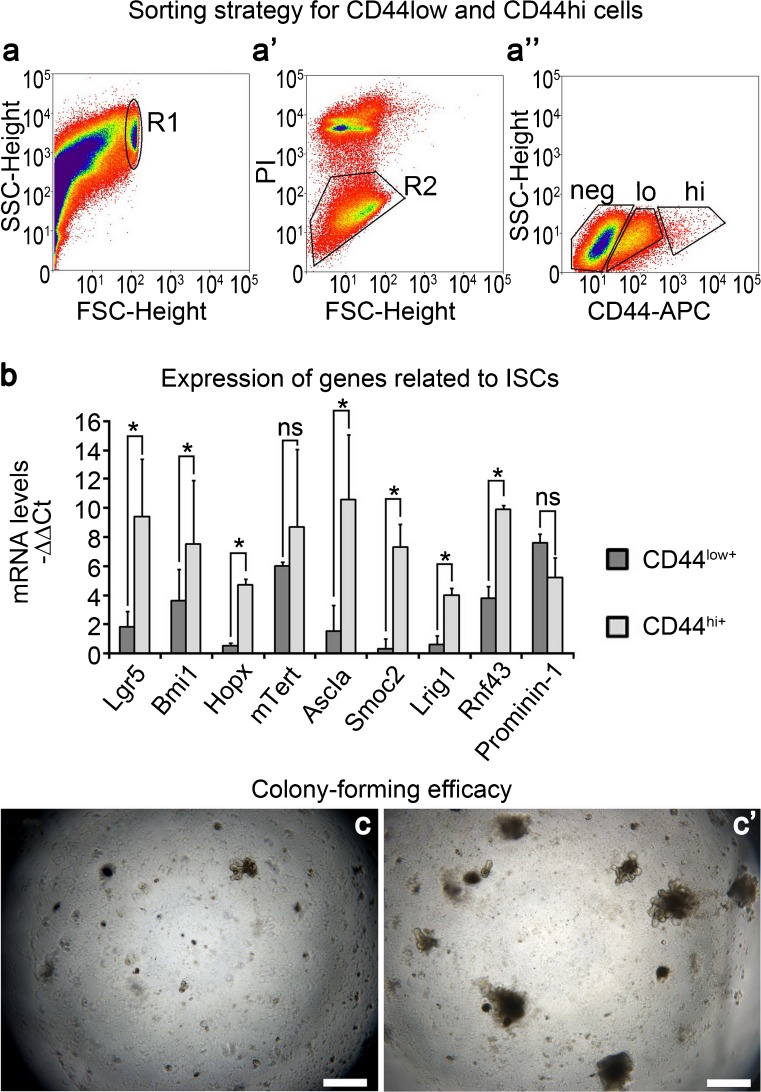
CD44^+^ ISCs resemble CBC stem cells. **a–a’’** Strategy for sorting CD44^-^, CD44^low+^ and CD44^hi+^ subpopulations by using the FACS technique (*R1* determining the cell-zone, *R2* determining viable cells, *PI* propidium iodide, *APC* allophycocyanin). **b** Semi-quantitative reverse transcription (RT) followed by the polymerase chain reaction (PCR) for ISC-related gene-expression in sorted cells. Fold expression values were normalized to the CD44^-^ group. Data represent means ± SD of six independent measurements (*n* = 6). *Bars* indicate value of SD. The paired *t*-test was used for data analysis. *P ≤ 0.05 represents high significance (CD44^hi+^ group versus CD44^low+^ group); ^$^ *P* ≤ 0.05 represents low significance (CD44^hi+^ group versus CD44^low+^ group); *ns* represents no statistic differences between the CD44^hi+^ group and CD44^low+^ group. *P*-values for *Lgr5*, *Bmi1*, *Hopx*, *mTERT*, *Ascl2*, *Smoc2*, *Lrig1*, *Rnf43* and *Prominin-1* are respectively 0.016, 0.036, 0.010, 0.844, 0.007, 0.001, 0.041, 0.005 and 0.859. **c**, **c’** Colony-forming efficacy of CD44^low+^ and CD44 ^hi+^ cells in 3D-culture system for 14 days. **c** CD44^low+^ group. **c’** CD44^hi+^ group. Magnification ×40. *Bars* 500 μm

When analyzing the purity of the microbead-sorted CD44^+^ crypt cells, we found that these cells could be classified into two subpopulations: CD44 high-positive (termed CD44^hi+^ below) and CD44 low-positive (termed CD44^low+^ below; Fig. 2a’’’’). Since the above results indicated that a population of ISCs was present in the CD44^+^ crypt cells, we speculated that CD44^hi+^ crypt cells were more similar to ISCs than CD44^low+^ cells. To test this hypothesis, we sorted these two subpopulations by using the FACS technique (Fig. 3a–a’’). Then, semi-quantitative RT-PCR analysis was used to compare the expression of genes related to ISCs between these two pools of cells, with the CD44^-^ population being set as the internal control. Relevant results showed that the ISC-related genes, including *Lgr5*, *Bmi1*, *Hopx*, *Ascl2*, *Smoc2*, *Lrig1* and *Rnf43*, were significantly up-regulated with regard to their expressions in CD44^hi+^ crypt cells, whereas no significant differences were found in the expression of each of *mTERT* and *Prominin-1* between these two populations (Fig. 3b). On this basis, we tried to compare the organoid-forming capabilities between these two cell-populations. The results showed that the CD44^hi+^ crypt cells were capable of forming organoids during a period of 2 weeks culturing, whereas most of the CD44^low+^ cells could only form cell clusters in vitro (Fig. 3c, c’). All these data indicated that CD44^hi+^ crypt cells resembled CBC stem cells, whereas CD44^low+^ cells were similar to TA progenitors.

### Epithelial homeostasis in CD44^+^ ISC-derived organoids

**Fig. 4:**
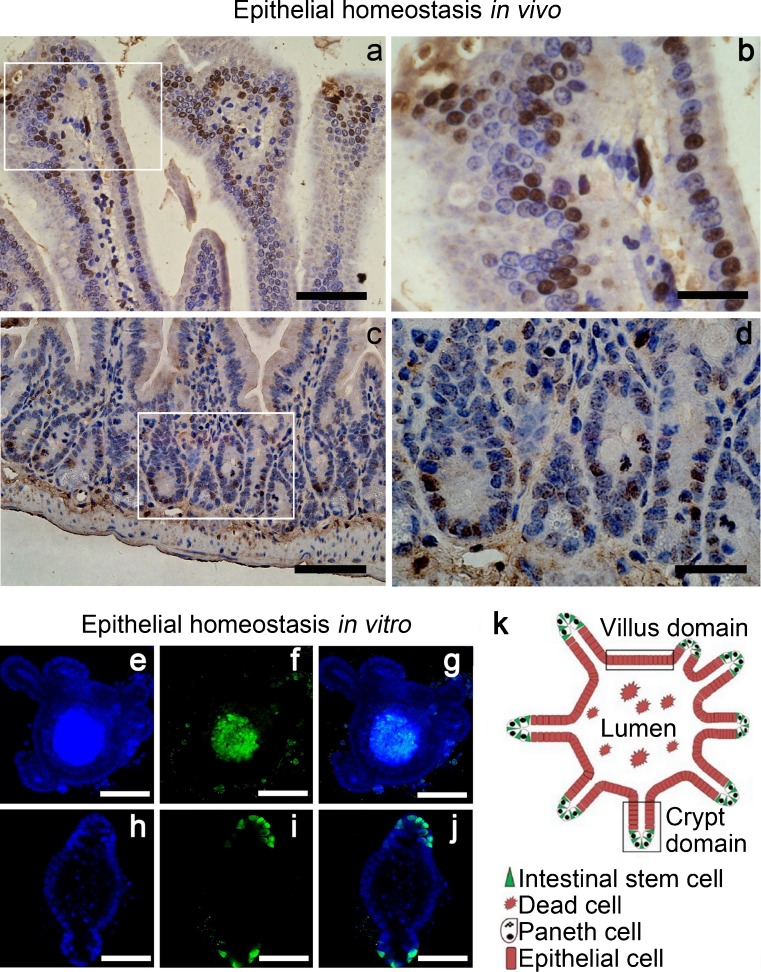
Epithelial homeostasis in CD44^+^ ISC-derived organoid. **a**, **b** TUNEL staining for apoptotic cells in normal epithelium. **c**, **d** IHC staining of Ki67 for proliferative cells within normal epithelium. **a**, **c** Magnification ×200. *Bars* 100 μm. **b**, **d** Magnification ×400. *Bars* 50 μm. **e–j** Epithelial homeostasis in vitro. **e–g** TUNEL staining for apoptotic cells in CD44^+^ ISC-derived organoid. **e** DAPI staining (*blue*) for nuclei. **f** dUTP-FITC (*green*) for apoptotic cells. **g** DAPI image merged with dUTP-FITC image. Magnification ×100. *Bars* 200 μm. **h–j** ICC staining for proliferative cells in CD44^+^ ISC-derived organoid. **h** DAPI staining for nuclei. **i** Ki67-FITC for proliferative cells. **j** DAPI image merged with Ki67-FITC image. Magnification ×200. Bar 100 μm. **k** Representation of an organoid

The growing mechanism of Lgr5^+^ CBC stem cells has been well investigated (Sato et al. 2009). In the present study, the apoptotic and proliferative activities were analyzed to determine the specific homeostasis in CD44^+^ ISC-derived organoids. In vivo, the apoptotic cells were mainly located at the top of villi, whereas the proliferative cells were arranged in the crypt domain (Fig. 4a–d). The organoids shared similar characteristics. As shown in Fig. 4e–g, the TUNEL analysis suggested that most of the apoptotic cells were located in the lumen of organoids, indicating that the apoptotic cells were shed from the villus domain. In addition, most Ki67-positive cells were merely located within the crypt domain indicating that the development of organoids was driven by the proliferation of ISCs and by the commitment of TA progenitors into functional epithelial cells (Fig. 4h–j). In contrast to the typical structure of the villus-crypt axis in vivo, the organoids contained finger-like crypts and flat villi (Fig. 4k), as formed naturally and escaped from the compression caused by tissue-tensions in vivo. Taken together, our present findings suggest that CD44^+^ ISC and CBC stem cells share similar homeostatic activities when they grow into organoids.

### Radiation-induced crypt death can be delayed by co-culturing with MSCs

**Fig. 5:**
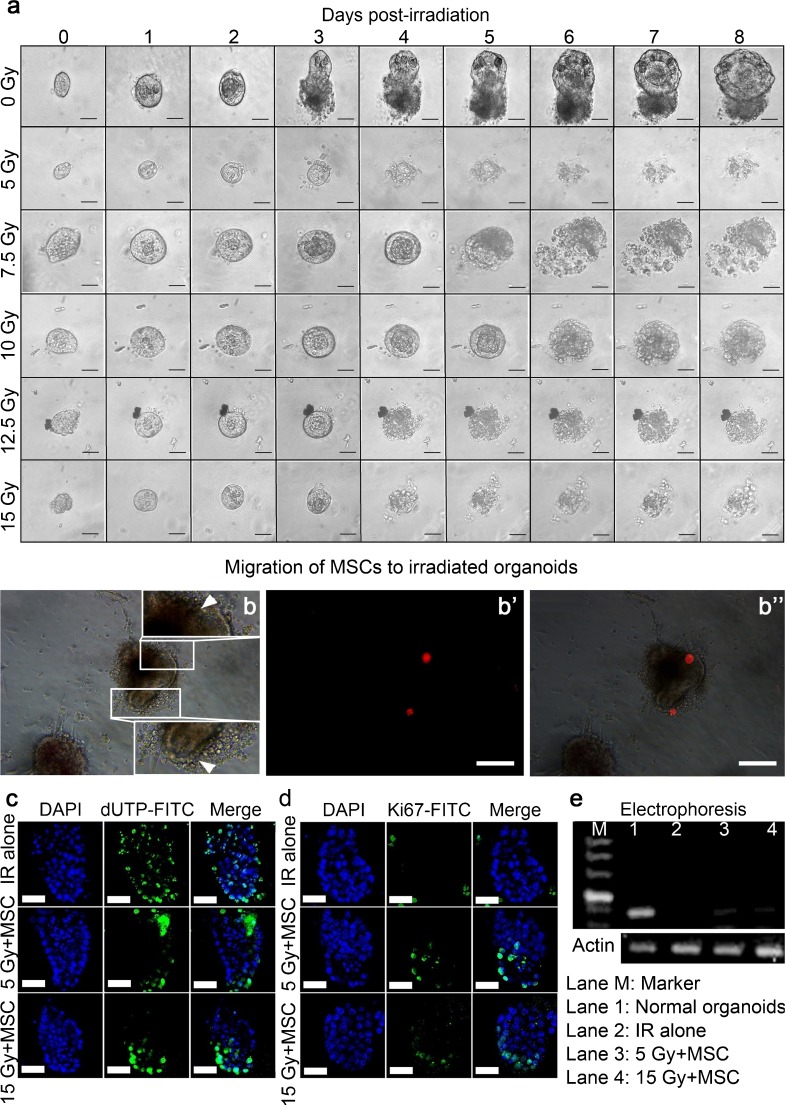
Mensenchymal stem cells (*MSCs*) delay radiation-induced crypt death. **a** In vitro growth of crypt fragments during a period of 8 days post-irradiation. *Numbers* represent days post-irradiation. Magnification ×400. *Bars* 50 μm. **b–b’’** Chemotactic property of MSCs in vitro. In a transwell experiment, MSCs were plated on the upper mesh of 12-μm bore diameter. The organoids were plated on the lower plate by using Matrigel. At 48 h after co-culturing, MSCs migrated to the irradiated organoids. **b** Phase-contrast imaging for organoids (*white arrowheads* infiltrated MSCs). **b’** Fluorescent imaging for MSCs labeled with CM-Dil (a lipophilic marker). **b’’** Blended image. Magnification ×200. *Bars* 100 μm. **c** TUNEL staining for apoptotic cells within organoids. **d** ICC staining of Ki67 for proliferative cells within organoids. **c**, **d** Magnification ×630. *Bars* 20 μm. **e** RT-PCR for *Cd44v6* expression in organoids. Electrophoresis in 1.5 % agarose gel (*M* marker, *Lane 1* normal group, *Lane 2* irradiation alone [*IR*] group, *Lane 3* 5 Gy + MSC group, *Lane 4* 15 Gy + MSC group). Actin was used for the internal control

Intestinal epithelium represents a tissue of high radiosensitivity, which is partially attributable to some fast-cycled ISCs (Metcalfe et al. 2014). As mentioned above, ISCs are only located in the crypts and can drive the development of organoids in vitro. On this basis, we investigated the inhibitory effects of ionizing irradiation on organoid formation. Anoikis in single-isolated ISCs affects their abilities to form organoids in vitro (Sato et al. 2009). To avoid this, we used crypt fragments. A single dose of 5 Gy, 7.5 Gy, 10 Gy, 12.5 Gy, or 15 Gy was separately administered to the freshly isolated crypt fragments. Strikingly, the relevant results showed that the crypt fragments halted their growth and loosened their structures around 2 to 4 days post-irradiation (Fig. 5a), indicating that ionizing-irradiation-induced crypt death occurred irrespective of the radiation doses and that all supplements in the medium were insufficient to improve the resistance of crypts to ionizing irradiation.

Next, we tried to determine whether the MSCs were capable of limiting apoptosis in irradiated organoids, as our previous work had demonstrated that MSC infusion could promote epithelial regeneration in a rodent model of radiation-induced intestinal injury (Chang et al. 2013). To this end, the freshly isolated crypt fragments were induced to form organoids during the first 7 days (by using a plating density of 500 crypt fragments per well in a 24-well plate). Then, the organoids were separately irradiated at 5 Gy and 15 Gy and were then co-cultured immediately with human MSCs in a transwell system (bore diameter of upper mesh: 8 μm) at a ratio of 1:100. At 48 h post-irradiation, some MSCs were located next to the irradiated organoids (Fig. 5b–b’’) and the crypt domain in the organoids possessed fewer apoptotic cells than those receiving ionizing radiation alone (Fig. 5c). Meanwhile, a certain number of proliferative cells still existed in the irradiated crypt fragments, when co-culturing with MSCs (Fig. 5d, Supplemental Fig. S2). However, the proliferative activity within these crypts co-cultured with MSCs was decreased, whereas the apoptotic activity was swiftly increased at 72 h post-irradiation (data not shown), indicating that these crypts would still undergo radiation-induced death in spite of MSC intervention. Moreover, the proliferation in the crypt domain of irradiated organoids appeared to be related to the number of MSCs in the co-culture system (Supplemental Fig. S3). In addition, the MSCs in the co-culture system showed their proliferative potential together with morphological alterations from spindle-like shapes to elliptical shapes (Supplemental Figs. S4, S5). Proliferative cells in the crypt domain are characterized by their expression of *Cd44 variant 6* (termed *Cd44v6*; Schmitt et al. 2015). Our findings showed that, upon intervention by MSCs, the expression of *Cd44v6* still existed in crypt fragments at 48 h post-irradiation (Fig. 5e). By contrast, no *Cd44v6* was detected in irradiated crypts, reflecting that ISCs and TA progenitors were nearly depleted. Although the exact duration of the delay in radiation-induced crypt death caused by MSC intervention was difficult to define, our results indicated that more irradiated crypts survived at 48 h upon co-culturing with MSCs than without them.

## Discussion

In this study, we successfully isolated CD44^+^ ISCs from the small intestine of C57BL/6 mice and confirmed the “stemness” of the CD44^+^ ISCs by analyzing their capabilities including their self-expansion and multiple differentiation into functional cells within the epithelium in vitro. These results demonstrated that CD44 can be used for marking ISCs.

To our knowledge, a controversy concerning the location of ISCs has persisted for several years, because ISCs at different sites possess distinct characteristics, including their numbers in crypts, their radiosensitivities and the mechanism for parental DNA segregation into progeny during division (Schepers et al. 2011; Potten et al. 2002). Despite this, we confirmed that the ISCs are located within the crypt. The present results show that cells strongly positive for CD44 are mainly found within the crypts, both in vitro and in vivo, at the putative sites in which ISCs are located, including the crypt basement and the 4+ position of the crypt. On this basis, we further attempted to isolate the CD44^+^ cells from the intestinal epithelium in order to seek evidence indicating that ISCs do indeed exist in this cell population.

Nowadays, the FACS technique is widely applied to sort CBC stem cells from C57BL/6^lgr5-eGFP-IRES-CreERT2^ reporter mice according to the gradient expression of the *Lgr5* gene in crypts (Sato et al. 2009). To sort ISCs from wild-type mice, several strategies have been explored, including the use of a single antigen, such as CD24 (von Furstenberg et al. 2011) or EphB2 (Sato et al. 2011b) and combined antigens, such as CD24/Sox9 (Gracz et al. 2010), CD24/CD44 (Gracz et al. 2013) and CD24/CD44/CD166/GRP78/c-Kit (Wang et al. 2013). In the present study, the marker of CD44 was used to isolate ISCs by means of the microbead-based sorting technique. As indicated previously, the ISCs undergo apoptosis shortly after being detached from their niches, termed anoikis and the ROCK inhibitor, Y-27632 dihydrochloride, can be used to prevent anoikis in ISCs (Sato et al. 2009). For the FACS technique, the protection of ISCs against apoptosis by the supplementation of sufficient Y-27632 dihydrochloride in a sheath of liquid is difficult. In contrast, a relatively small system for ISC sorting can be achieved by using the microbead-based technique, ensuring that adequate Y-27632 dihydrochloride covers the entire sorting process and thus maintains the viability of ISCs. However, the microbead-based sorting technique is not as good as the FACS technique in distinguishing the expression levels of putative markers related to ISCs. Moreover, the FACS technique allows a combination of several markers during one single sorting process, which ensures the precise identification of ISCs.

The present study has demonstrated that ISCs exist in the CD44^+^ population through using a microbead-based technique. However, when analyzing the purity of the sorted cells, we also found that the CD44^+^ population can be classified into two subpopulations: CD44^hi+^ and CD44^low+^. Further analysis of the distribution of CD44^+^ cells in the epithelium revealed that the CD44^hi+^ cells are apt to be CBC stem cells rather than reserve ISCs at the 4+ position of the crypt. First, both the *Cd44* gene and *Lgr5* gene are targets of the Wnt/β-catenin signaling pathway (Barker et al. 2007; Wielenga et al. 1999). Second, the proliferation of reserve ISCs is independent of the activating Wnt/β-catenin signaling pathway (Barker et al. 2012). Third, nearly 10 % of Lgr5^+^ ISCs are located at the 4+ position of the crypt partially accounting for the CD44^hi+^ cells at this position (Barker et al. 2007). In our study, quantitative RT-PCR analysis also revealed that CD44^hi+^ cells express high levels of ISC-related genes, such as *Lgr5*, *Bmi1*, *Hopx*, *Ascl2*, *Smoc2*, *Lrig1* and *Rnf43*. Several of these genes, including *Lgr5*, *Ascl2*, *Smoc2* and *Rnf43*, have been reported to be highly expressed by CBC stem cells (Barker et al. 2007; Schepers et al. 2011; Koo et al. 2012; Muoz et al. 2012). Concerning the genes for marking the reserve ISCs, including *Bmi1*, *Lrig1*, *Hopx* and *mTERT*, CBC stem cells also keep their expression of these genes (Muoz et al. 2012). However, our results have shown that the *mTERT* gene does not up-regulate its expression in CD44^hi+^ cells compared with CD44^low+^ cells. As reported by Breault et al. (2008), only one single cell positive for mTERT can be found within ∼157 crypts, indicating the rare expression of the *mTERT* gene by crypt cells. Moreover, the *mTERT* gene is also expressed by TA progenitors (Barker et al. 2012), partially accounting for the lack of a significant difference between CD44^hi+^ cells and CD44^low+^ cells in their *mTERT* expression. Similarly, no difference has been found between CD44^low+^ cells and CD44^hi+^ cells with regard to the expression of *Prominin-1*, which has been demonstrated to be highly expressed by TA progenitors (Snippert et al. 2009). Moreover, CD44^low+^ cells seldom expand their numbers to develop into organoids in vitro, as compared with CD44^hi+^ cells. This evidence suggests that TA progenitors mainly occur in the CD44^low+^ subpopulation. Thus, the sorting of CD44^+^ cells can harvest CBC stem cells, which mainly exist in the CD44^hi+^ subpopulation.

In adult intestine, CBC stem cells are responsible for maintaining epithelial homeostasis mainly under the control of signaling pathways, including Wnt/β-catenin, Ras/Raf/Mek/Erk, Notch and BMP/Smad (Sato and Clevers 2013). After their commitment into TA progenitors, these progenitors are devoted to differentiating into functional epithelial cells along the villus-crypt axis for replenishing dead cells. In the same way, the CD44^+^ ISC-derived organoids also retain the mechanism by which the proliferative cells within the crypt domain drive epithelial homeostasis. Within the CD44^+^ ISC-derived organoids, the proliferative cells are exclusively arranged in the crypt domain, whereas apoptotic cells are shed into the lumen. However, the organoids seem to be radiosensitive in vitro.

As described above, the CD44^+^ ISCs resemble CBC stem cells. However, the CBC stem cells are radiosensitive. Previous in vivo data have shown that a dose of only 0.01 Gy irradiation can initiate apoptosis in 10 % of CBC stem cells, whereas the intestine can tolerate the depletion of such radiosensitive CBC stem cells by presenting no alteration in epithelial architecture (Metcalfe et al. 2014; Zhu et al. 2013). When the intestine receives 6 Gy to 12 Gy, epithelial regeneration depends on the action of reserve ISCs converting into CBC stem cells, indicating the indispensability of CBC stem cells for epithelial regeneration (Metcalfe et al. 2014). In contrast to the above in vivo results, we found that the crypt fragments, receiving doses between 5 Gy and 15 Gy, will be dead within ∼4 days, implying a rapid depletion in CBC stem cells and an absence of activated reserve ISCs. The reason probably lies in the presently used 3D-culture system, which lacks essential factors for maintaining the growth of reserve ISCs (Sato et al. 2009), because these ISCs are independent of Wnt-driven proliferation (Barker et al. 2012). Alternatively, the niches for ISCs are nearly destroyed following irradiation, because the mature epithelial cells are even more sensitive to ionizing irradiation than CBC stem cells (Hua et al. 2012). However, recent in vivo data suggest that Paneth cells, the niche cells for ISCs, will disappear from crypts when irradiated at a dose of 15 Gy (Metcalfe et al. 2014; Sato et al. 2011a, 2011b). The present results indicate that crypt death occurs irrespective of irradiating doses.

In order to protect the crypts against radiation-induced death, we attempted to culture MSCs with irradiated organoids, as our previous data had suggested that an infusion of MSCs could accelerate epithelial regeneration through increasing the number of reserve ISCs within irradiated intestine (Chang et al. 2013). The present results show that, although no preservation of architecture and ultimate death are noticed in irradiated organoids, the crypts in those irradiated organoids co-cultured with MSCs still present fewer apoptotic cells, whereas more proliferative cells are found within the crypt domain compared with the controls. Moreover, *Cd44v6* expression is maintained in these irradiated organoids at 48 h after co-culturing with MSCs. As described above, *Cd44v6* is uniquely expressed by proliferative cells within crypts, which undoubtedly contain ISCs and TA progenitors (Schmitt et al. 2015). In addition, recent data have suggested that the CD44 protein is capable of increasing the activity of the Wnt/β-catenin signaling pathway through the regulation of the localization of low-density lipoprotein receptor-related protein 6 (LRP6) in the cell membrane (Schmitt et al. 2015). LRP6 is well documented as playing a critical role in the proliferation of ISCs. Mechanistically, the Wnt3 stimulates ISC proliferation mainly through its co-instantaneous binding to the LRP6 receptor and the extracellular end of the Frizzled receptor, an action that leads to the intracytoplasmic accumulation of β-catenin for pulsing on the expression of the *c-Myc* gene (Sato and Clevers 2013; Bettess et al. 2005). On this basis, we speculate that the down-regulation of *Cd44v6* will hamper the Paneth cell (a source of Wnt3)–initiated proliferation of ISCs in irradiated organoids because of the low activity of the Wnt/β-catenin signaling pathway, which subsequently results in the down-regulation in *Lgr5* expression (Sato et al. 2011a, 2011b). On this basis, the R-spondin1 hardly amplifies the responses of ISCs to Wnt3 -initiated proliferation because of the lack of Lgr5 receptors on the cell membrane (Yui et al. 2012; Binnerts et al. 2007). In contrast, when co-cultured with MSCs, irradiated organoids can preserve their *Cd44v6* expression, indicating that proliferative cells still exist within irradiated crypts. However, epithelial homeostasis in irradiated organoids cannot be maintained by co-culturing with MSCs. To some extent, the MSCs competitively absorb nutrients from the medium for their self-expansion; this is also harmful for the growth of irradiated organoids.

In this study, the chemotactic property of MSCs was also investigated through transwell experiments. MSCs are observed around organoids at 48 h post-irradiation, compared with the controls (data not shown). Additionally, the MSCs present pie-like shapes in the medium for organoid growth, whereas their self-expanding ability is maintained in this medium. However, we have not evaluated any alterations in the properties of MSCs, such as their phenotype and multi-lineaged differentiation. On this basis, whether the medium for organoid growth can alter the secreting profile of MSCs remains unknown. Nevertheless, MSCs are known to be potent in preventing cell apoptosis through autocrine/paracrine actions. For example, cytokines including hepatocyte growth factor (HGF), prostaglandin E2 (PGE2), insulin-like growth factor-1 (IGF-1) and basic fibroblast growth factor (bFGF), are reported to be capable of enhancing the resistance of ISCs to ionizing irradiation predominantly through increasing the activity of the PI3K/Akt signaling pathway (Binnerts et al. 2007; Todaro et al. 2014; Tessner et al. 2004; Qiu et al. 2010). Collectively, the present data suggest that MSCs can delay crypt death but cannot maintain epithelial homeostasis. The detailed action of MSCs in delaying crypt death deserves further investigation.

In conclusion, CD44 can be used for marking ISCs and CD44^+^ ISCs resemble CBC stem cells. To some extent, MSCs can increase the radioresistance of the epithelium in vitro.

## Electronic supplementary material

Below is the link to the electronic supplementary material.

ESM 1 (DOCX 4009 kb)
