# Supplementary material for: Mensenchymal stem cells can delay radiation-induced crypt death: impact on intestinal CD44+ fragments
Source: Cell Tissue Res. 2015 Nov 27;364:331–44. doi: 10.1007/s00441-015-2313-6 (PMC4846698; doi:10.1007/s00441-015-2313-6)
Supplement: Supplementary file 1 — (DOCX 4009 kb) [file 441_2015_2313_MOESM1_ESM.docx]

**Title: CD44: A candidate for marking intestinal stem cells**

Author list: Peng-Yu Chang, Xing Jin, Yi-Yao Jiang, Li-Xian Wang,

Jin Wang* and Yong-Jun Liu*

**Supplemental Figure S1**

Differentiation of CD44+ ISC into functional epithelial cells by IF staining.

Villin: Absorptive cell; Muc2: Goblet cell; Chr-A: Endocrine cell; Lysozyme C: Paneth cell. Magnification at 200 ×; Bar: 20 μm. White arrow: Positive cells within CD44+ ISC-derived organoids.


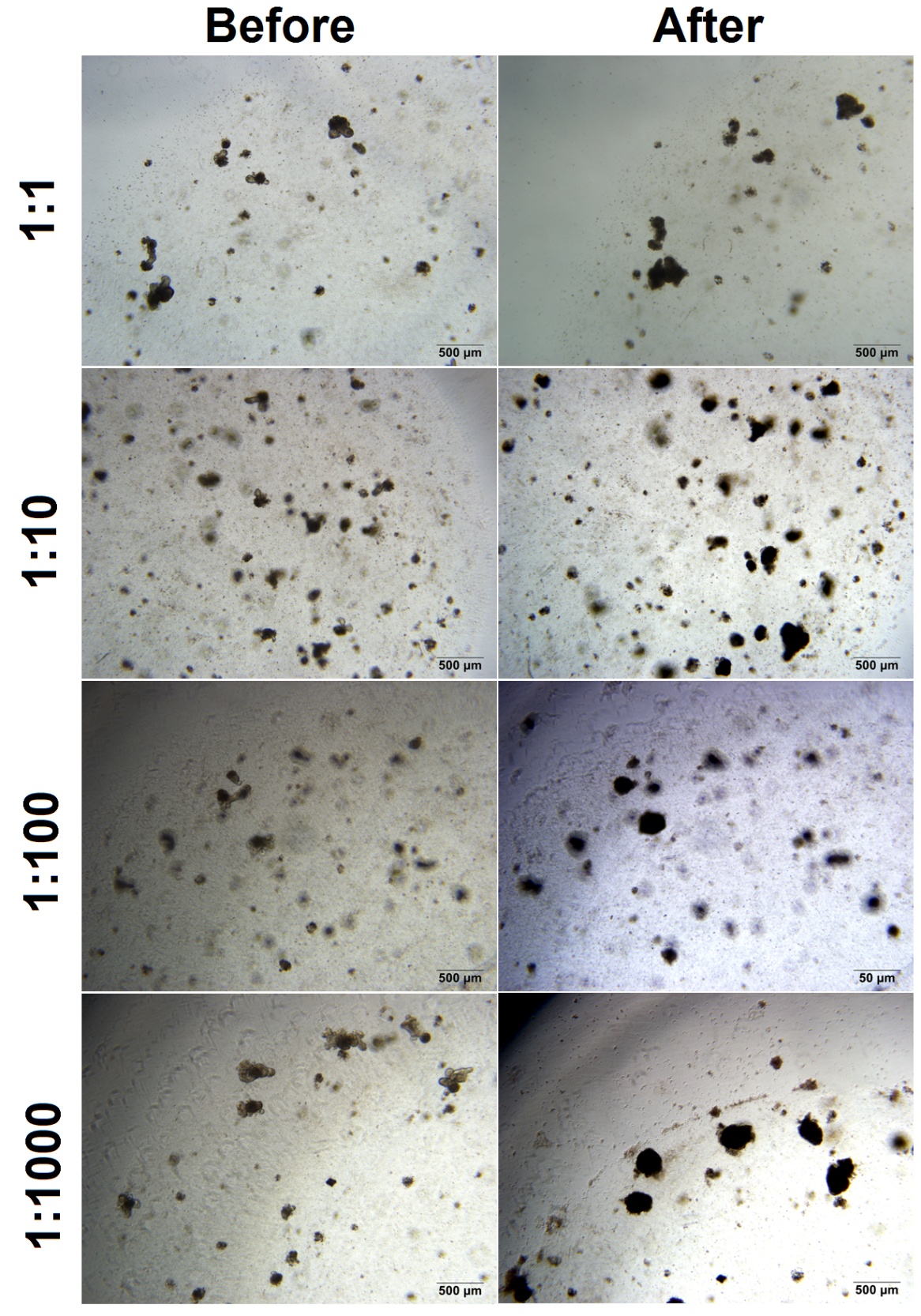


**Supplemental Figure S2**

Ultimate death of irradiated organoids in spite of co-culturing with MSCs.

The number listed in this figure represents the ratio of oganoids versus MSCs.

Magnification at 40 ×; Bar: 500μm.


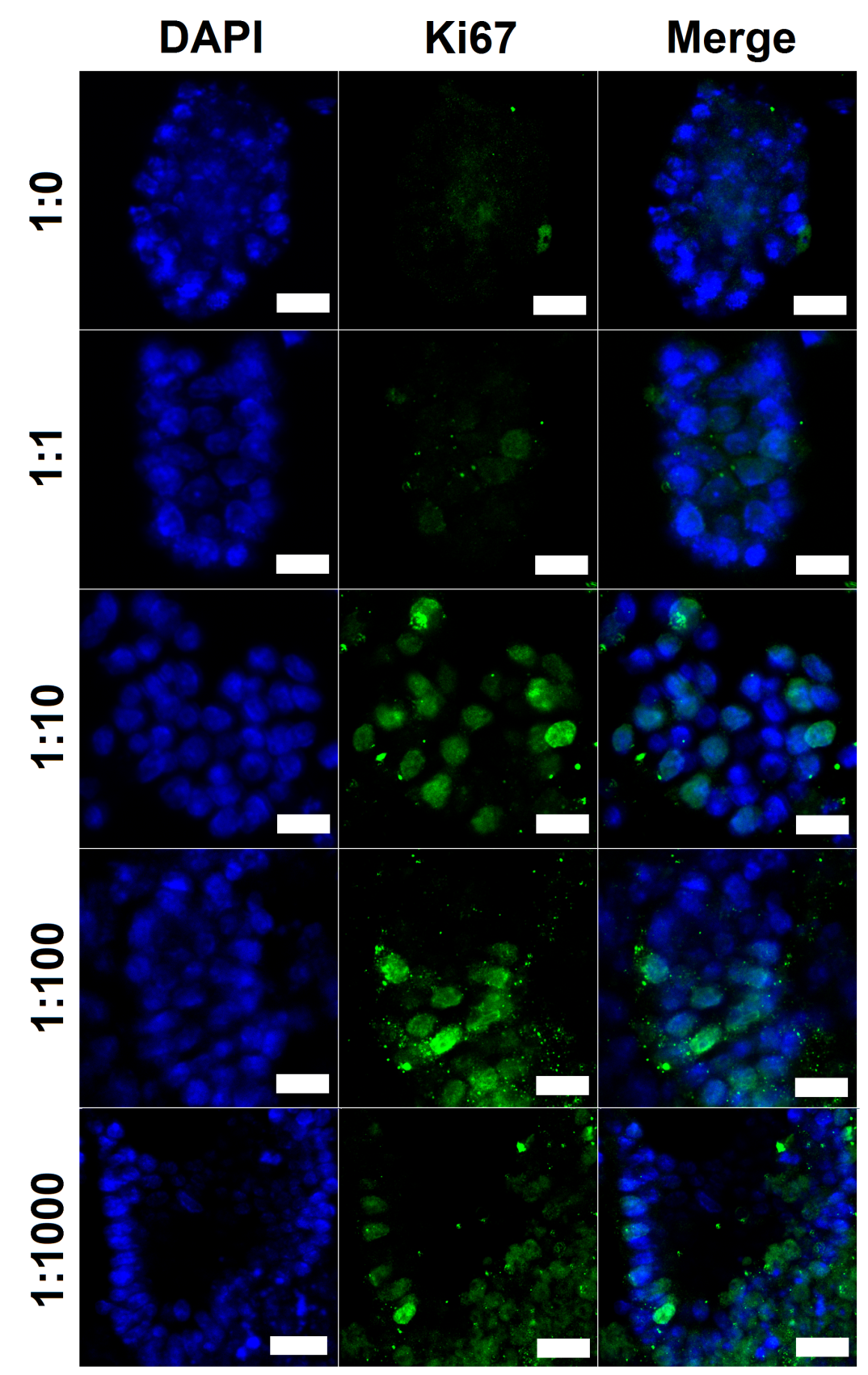


**Supplemental Figure S3**

IF staining for analyzing the relevance between MSCs number in maintaining the proliferative status of crypt cells.

The number listed in this figure represents the ratio of oganoids versus MSCs.

Magnification at 630 ×; Bar: 20 μm.


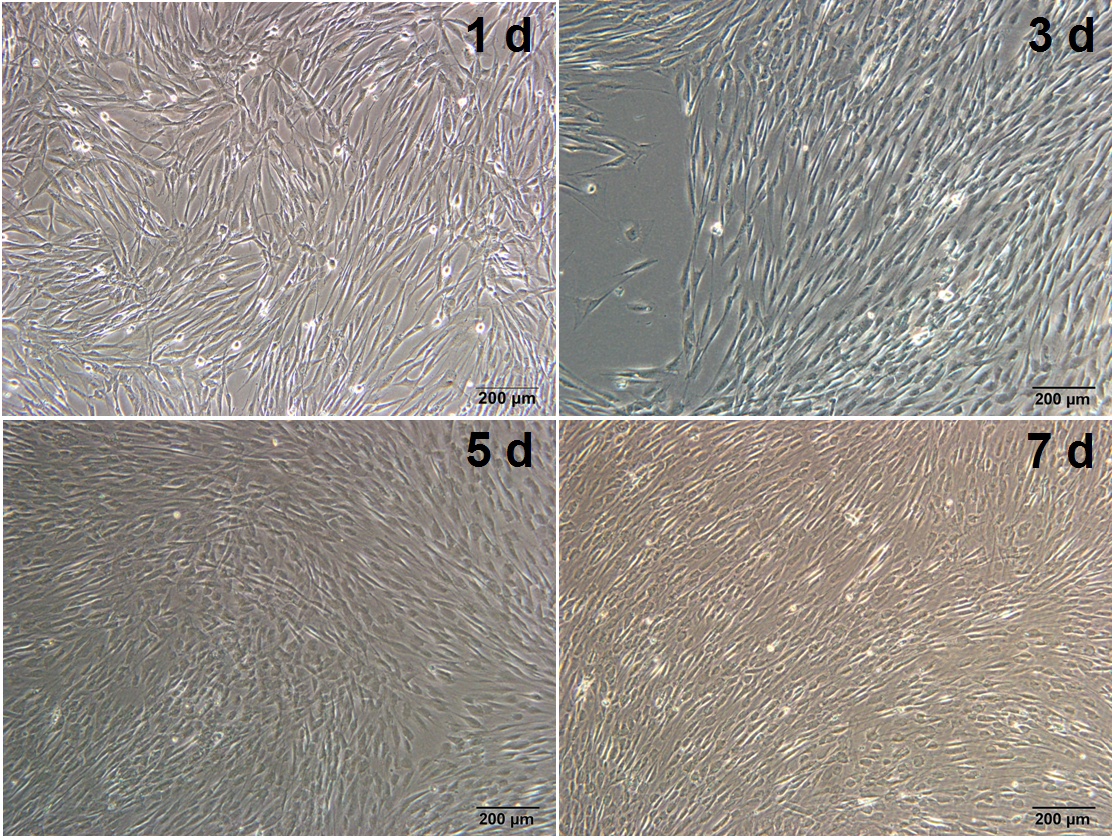


**Supplemental Figure S4**

Morphological alteration of MSCs in the medium for organoid growth.

The number represents the day after culturing. Magnification at 100 ×; Bar: 200 μm.


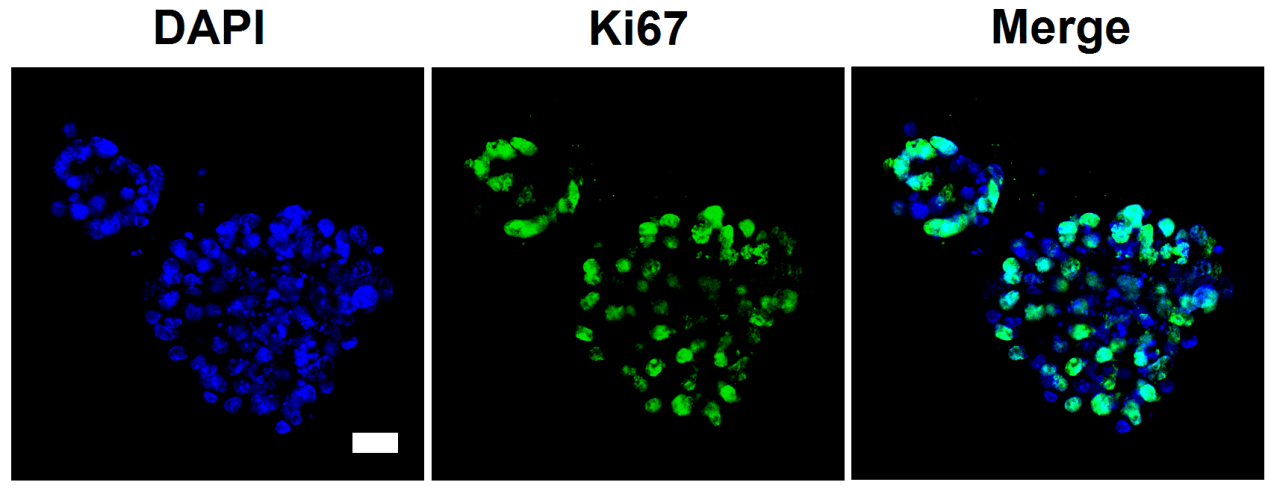


**Supplemental Figure S5**

Self-expanding ability of MSCs in medium for organoid growth by IF staining.

Magnification at 200 ×; Bar: 20 μm.

**Supplemental Table S1**

| Gene name | Sequence (5’ – 3’) | Product size (bp) |
| --- | --- | --- |
| Lgr5 | F: GGTGGGACCACGATTCTCTG | 454 |
|  | R: GCAGAGGCGATGTAGGAGAC |  |
| Bmi1 | F: GCAGAAGTTTTGGGAACCCTG | 295 |
|  | R: AGTGCCTCAAACGCACTCTC |  |
| Hopx | F: GGAGGAGCAGACGCAGAAAT | 213 |
|  | R: AGCAGGACAGCAAAACAATG |  |
| mTERT | F: CTTGCTGCTGGACACTCAGA | 613 |
|  | R: TAGCGGAAGGAGACAGGGTT |  |
| Lrig1 | F: GTGCACGGGTCTGTAGTTGA | 217 |
|  | R: GAGGCAGAGCTAACCCTCAC |  |
| Ascl2 | F: AAGCTTGGTCCGGTTCTTCA | 253 |
|  | R: CCAGTCAAGGTGTGCTTCCA |  |
| Smoc2 | F: GTCACACAGGCACTAGCTCA | 241 |
|  | R: CCAGGCACTCAGAAGTGAGG |  |
| Rnf43 | F: CGGAGCCGTACTGGGTTAAG | 409 |
|  | R: GGGAGCAGAAGATGGCCTTT |  |
| Prominin-1 | F: GCCCTGAGGATGGTACTTTGA | 254 |
|  | R: AGCCCCAGGAGTGTTATGGA |  |
| Cd44v6 | F:CATCGAGAAGAGCACCCCAG | 119 |
|  | R:GACTCTGTGTGGTGGCCAAG |  |
| Actin | F: TGGAGCAAACATCCCCCAAA | 315 |
|  | R: AGACCTGGGCCATTCAGAAA |  |
